# Long Term Effectiveness of ESWT in Plantar Fasciitis in Amateur Runners

**DOI:** 10.3390/jcm11236926

**Published:** 2022-11-24

**Authors:** Joanna Kapusta, Marcin Domżalski

**Affiliations:** 1 Department of Internal Diseases, Rehabilitation and Physical Medicine, Medical University of Łódź, 70-445 Lodz, Poland; 2 Department of Orthopaedics and Trauma, Veteran’s Memorial Hospital, Medical University of Lodz, Zeromskiego 113, 90-549 Lodz, Poland

**Keywords:** extracorporeal shock wave, heel spur, plantar fascia, rehabilitation, sports medicine

## Abstract

Background: Shock wave therapy is one of the modern methods of treatment used to treat diseases of muscles, tendons, and entheses in orthopedics, as well as in sports medicine. The therapy is increasingly used in the treatment of plantar fasciitis—a disease that is very difficult and burdensome to treat. Where basic conservative treatment for heel spurs fails, the only alternative consists of excision of the bone outgrowth, and shock wave therapy: a modern, minimally invasive, and relatively safe method. The aim of the study was to determine the long-term effectiveness of extracorporeal shock wave therapy in the treatment of painful ailments occurring in the course of plantar fasciitis in amateur runners. Materials and methods: The study includes a group of 39 men and women, aged 34–64 (mean age 54.05 ± 8.16), suffering from chronic pain in one or both feet, occurring in the course of plantar fasciitis. The patients had to meet five criteria to qualify for the study. The group was divided into two subgroups: those who had not undergone other physiotherapeutic procedures prior to the extracorporeal shock wave therapy (ESWT-alone; 23 people), and those who had received other procedures (ESWT-plus; 16 people). The therapy was performed using extracorporeal shock wave (ESWT). No local anesthesia was used. The effectiveness of the extracorporeal shock wave therapy was evaluated using the visual analogue scale of pain (VAS), Modified Laitinen Pain Index Questionnaire, the AOFAS scale (American Orthopedic Foot and Ankle Society), and a survey questionnaire consisting of 10 questions concerning metrics and subjective assessment of the effects of therapy. The interview was conducted before ESWT, and again five years later. Results: The use of extracorporeal shock wave therapy reduced the intensity and frequency of pain, and improved daily and recreational activity. Moreover, a reduction in the level of pain sensation on the VAS scale and pain symptoms during walking was demonstrated. More favorable results were obtained in the ESWT-plus group; however, the first effects were observed later than in the ESWT-alone group. Conclusions: Extracorporeal shock wave therapy is an effective form of therapy for amateur runners. It reduces pain associated with plantar fasciitis that amateur runners may experience at rest, while walking, and during daily and recreational activity.

## 1. Introduction

Plantar fasciitis with an accompanying heel spur is very burdensome and difficult to treat disease [1,2,3,4,5]. It most often arises as a result of degenerative changes of the proximal plantar fascia and the tissues surrounding the aponeurosis, occurring due to continuous irritation of the area and resulting micro-injuries [6,7].

The main symptom of plantar fasciitis is pain in the heel area; this worsens over time, increasingly occurring upon loading and eventually, even at rest. Redness and swelling are also observed in the heel. The risk of the disease is increased by being overweight, working a job that requires long periods of standing, lifting heavy objects, intensive running, and practicing jumping sports [6,7,8].

Conservative treatment consists of strengthening the long muscles of the foot, relieving the painful area with special orthopedic insoles that have an opening for the heel in the place corresponding to the presence of bone growth. Appropriate body weight should be maintained and prolonged overloading of the foot should be avoided. While pharmacotherapy, radiation with X-rays [9,10] and physical therapy can be used, this conservative treatment is very frequently insufficient, and the only alternative is surgery consisting of excision of bone spurs. As such, shockwave therapy is becoming increasingly popular among doctors [4,6,7,8,9].

Shock wave therapy is a modern method based on the application of mechanical pressure waves directly to the affected tissues. Although it was initially used for crushing inoperable kidney stones, it is increasingly used in the treatment of lesions located within the musculoskeletal apparatus [1,2,3].

The mechanical waves can be generated by extracorporeal shock wave therapy (ESWT) or radial shock wave therapy (RSWT) [1,2,4]. An extracorporeal shock wave is characterized by deep penetration, a short pulse rise time with the steepness of the wave formed in the tissue, a frequency within the range of 1–22 Hz, as well as a very high energy of generated pulses, reaching even 120 MPa within the treated location [11,12,13,14]. The extracorporeal shock wave treatment has a very intense impact, and therefore local anesthesia is very often necessary during the procedure [12,13,14]. Radial shock wave therapy (RSWT), in contrast, is characterized by lower parameters, lower impact force, and a smaller range of penetration. Despite this, the two wave types have very similar therapeutic effects [1,2,4,15,16].

The mechanism of shockwave functioning is not fully understood. Initially, it was believed that the therapy induced positive therapeutic effects due to a structural breakdown of cells at the microstructural level, resulting in the activation of tissue regeneration processes [3,12,17]. It is now known that, at energy levels below the tissue destruction level, the shock wave also causes a range of other tissue responses and metabolic effects. These changes can increase joint mobility, and result in long-term pain relief and the restoration of normal muscle tone. The principal effects observed during shock wave therapy include reduction of pain, elimination of the source of pain, reduction of muscle tension, and improvement of the function of tissue structures, as well as induction of congestion and activation of regenerative processes [1,4]. However, the shock wave can also cause adverse effects, such as reddening of the skin, hematomas, or local swelling [1,12,17].

### The Aim of the Study

The aim of the study was to determine the long-term effectiveness of extracorporeal shock wave therapy in the treatment of painful ailments occurring in the course of plantar fasciitis in amateur runners.

## 2. Materials and Methods

### 2.1. Characteristics of the Study Participants

A group of 48 consecutive patients with the diagnosis of plantar fasciitis were identified and examined. Of these, 39 met inclusion criteria and were enrolled in the study.

Inclusion criteria comprised the following: plantar fasciitis (with/without heel spur) confirmed by sonographic examination, prescribed shock wave therapy treatments, no participation in other physical therapy procedures during ESWT therapy, consent given by the subject. The exclusion criteria comprised any lesion or rupture of the plantar fascia found during sonographic examination, systemic inflammatory or autoimmune disorders, previous surgeries of the lower limbs, hereditary deformations of the skeleton, any other contraindications to participating in the study. Patients included in the study did not receive any physical therapy prior to inclusion.

Patient age, sex, involvement side, height, weight, and type of work performed were collected from medical records. The effectiveness of extracorporeal shock wave therapy (ESWT) was determined using the visual analogue scale of pain (VAS), Modified Laitinen Pain Index Questionnaire, AOFAS score (American Orthopedic Foot and Ankle Society) and a questionnaire about the subjective assessment of the effects of therapy.

Prior to participation, the patients were informed of the study objectives and how the study would be conducted, after which they provided their informed consent to participate in the study. The study was conducted according to the guidelines of the Declaration of Helsinki and approved by the Bioethics Committee of the Medical University of Lodz, Poland (approval number RNN/879/11/KB).

### 2.2. Study Program

After the initial qualification of patients for the study, in order to avoid bias, patients were randomly assigned to groups by a person from the research team who had no previous contact with qualified patients. The subjects were divided into two groups: the first group, ESWT-alone (23 people), comprised those who had not participated in other physiotherapeutic procedures before the commencement of ESWT therapy. The second group, ESWT-plus (16 people) comprised those who had participated in ultrasound and laser treatments before ESWT therapy.

All patients in the ESWT-plus group received ultrasound and laser treatments for two weeks before the shockwave therapy was started. The ultrasound was performed daily, for 5 min, using the following parameters: continuous mode, base frequency of 1 MHz to produce a deeper penetration, power of 2 W/cm^2^ into the areas of the painful heel and the myofascial junction at the dorsum of the heel. Laser therapy was also performed daily, after the ultrasound treatment, for 5 min. All patients were treated with laser at a power of 50 mW. The laser probe was applied to the areas of the painful heel, on the medial calcaneal area, and at the dorsum of the heel, for a total dose of 8 J/cm^2^ for 200 s. The selection of parameters during the study was based on previous studies and available literature [18].

The shock wave therapy was performed using extracorporeal shock wave (ESWT). The patients underwent four treatments separated by weekly breaks. Treatment parameters: applied 1000 beats/min at a power density of 0.25 mJ/mm^2^. Local anesthesia was not used during the therapy. When selecting the parameters during the study, previous studies and available literature were taken into account [19].

The extracorporeal shock wave penetrates much deeper than the radial shock wave, and is therefore more suitable for this treatment [20,21].

Laser therapy, ultrasound therapy, and ESWT were performed by using the BTL-5000 SWT Power extended version of device.

The patients were interviewed to determine pain symptoms resulting from the presence of plantar fasciitis and the impact of the disease on the activity of everyday life and motor activity using the VAS scale, Laitinen questionnaire, AOFAS score, and a questionnaire for evaluating the effects of therapy. The interviews took place only at two time points: the first was before the extracorporeal shock wave therapy was performed (December 2015 to March 2016), and the second was five years after the procedure to check whether the pain had reappeared (December 2020 to March 2021). For the second interview, the patients were contacted by telephone. The therapeutic effects of the therapy were analyzed in terms of the parameters studied and the level of satisfaction throughout the period under study.

### 2.3. Statistical Analysis

The statistical analysis was performed using STATISTICA PL 13.3 software (StatSoft Polska, Krakow, Poland) and the R environment. Variables measured are described based on mean and standard deviation (SD), while those involving positional measurements are given as median (Me), inter-quartile range (IQR), and minimum and maximum (Min–Max). For variables measured, only positional measures are provided. For non-measurable variables, the number of observations with a given feature variant (N) and the corresponding percentage (%) are given.

The normality of the variables was verified using the Shapiro–Wilk test. As their distribution was not normal, the non-parametric Mann–Whitney U test was used to compare the two independent groups. Two-way order ANOVA with repeated measurements was used to compare the groups with repeated measures (i.e., before and after treatment).

For the qualitative variables, the groups were compared with the chi-square test of independence. Additionally (where it was justified), the effect size was calculated using the form effect size measurement: $r=z/\sqrt{N}$ (where *z* is the value of the *z* statistic in the Wilcoxon pairwise test and *N* is the sample size). The effect is considered weak when *r* ∈ (0.10–0.40), average when *r* ∈ (0.40–0.60), and too strong when *r* ∈ 0.60 [22]. Statistically significant results were obtained with *p* < 0.05.

## 3. Results

### Evaluation of Basic Characteristics

Of the 39 patients included in the initial study, all 39 were included in the final follow-up. The group comprised 22 women (56.41%) and 17 men (43.59%), aged 34–64 (mean age 54.05 ± 8.16). Among the patients who underwent shock wave therapy, 23 (58.97%) had not previously undergone any rehabilitation procedures (ESWT-alone), while 16 (41.03%) had previously participated in other rehabilitation procedures (ESWT-plus).

The mean BMI (body mass index) value was 28.46 ± 3.92 kg/m^2^ (range 20.05 kg/m^2^ to 37.13 kg/m^2^). In half of the patients, BMI did not exceed 28.41 kg/m^2^ (IQR: 25.89–31.14 kg/m^2^). No statistically significant difference in BMI was found between the groups (*p* = 0.2925); however, almost 61% of the study group (ESWT-alone) were overweight, while 37.5% of the comparative group (ESWT-plus) were overweight. The characteristics of both groups in terms of sex, age, and BMI are presented in Table 1, Table 2 and Table 3. 

Table 4 presents the assessment of pain intensity by the two treatment groups according to the VAS scale, before and after therapy. No significant intergroup difference was observed before or after therapy (ESWT-alone vs. ESWT-plus: *p* = 0.9809 before therapy and *p* = 0.9200 after therapy); however, a significant intragroup reduction in pain over time was observed in both groups (*p* < 0.0001). In both groups, the obtained effect should be considered strong; however, it was slightly greater in the ESWT-plus group.

Table 5 presents the characteristics of the two treatment groups before and after therapy, with regard to pain intensity assessed according to the modified Laitinen scale. No significant intergroup differences were noted before or after therapy (ESWT-alone vs. ESWT-plus: *p* = 0.8840 before therapy and *p* = 0.9687 after therapy). Significant intragroup reductions in pain, measured on the Laitinen scale, were found in both groups over time (*p* < 0.0001). In both groups, the obtained effect should be considered strong, while it was slightly higher in the ESWT-plus group.

The following results were obtained from the questionnaire: the intensity of pain decreased in 91.31% of the respondents in ESWT-alone, and in 100% in ESWT-plus. A significant improvement in the frequency of occurrence of pain was achieved after therapy in ESWT-alone; only two respondents experienced frequent pains. In ESTW-plus, none of the subjects experienced frequent or continuous pain after the therapy. Both groups reported not needing to take painkillers after therapy. Finally, in both groups, 100% reported improvement in physical activity; however, partial limitation of physical activity was half as common in the ESWT-plus group after shock wave therapy.

Table 6 presents the AOFAS (total score) results before and after therapy. No significant intergroup differences were found before or after therapy (ESWT-alone vs. ESWT-plus: *p* = 0.9645 before therapy and *p* = 0.8380 after therapy), while a statistically significant (*p* < 0.0001) intragroup increase was observed in each group over time. In both groups, the obtained effect should be considered strong, while it was slightly higher in ESWT-plus.

Table 7 presents the pain assessment (AOFAS—Pain) results before and after treatment. No significant intergroup differences were found before or after therapy (ESWT-alone vs. ESWT-plus: *p* = 0.9535 before therapy and *p* = 0.7676 after therapy). Significant intragroup increases in AOFAS score were found in both groups over time (*p* < 0.0001). In both groups, the obtained effect should be considered strong, while it was slightly higher in the comparative group (with previous rehabilitation treatments).

Table 8 presents the function point scores (AOFAS—Function) before and after therapy. No significant intergroup differences were found before or after therapy (ESWT-alone vs. ESWT-plus: *p* = 0.9396 before therapy and *p* = 0.9574 after therapy); however, statistically significant intragroup increases in the AOFAS-function score were found in both groups over time (*p* = 0.0001 and *p* = 0.0013). In both groups, the obtained effect should be considered strong, while it was slightly higher in ESWT-alone.

Table S1 (Supplementary Materials) presents the time at which the first treatment effects were noted in the groups. A statistically significant difference was found between the groups, with the effects of therapy being observed earlier in the ESWT-alone group than in the ESWT-plus group (almost 70% of patients after the first treatment) (*p* = 0.0190).

In addition, in the ESWT-alone group, 91.30% of patients reported feeling more physically fit after the treatments; in contrast, in the ESWT-plus group, 100% reported an improvement in physical fitness. Tables S2 and S3 (Supplementary Materials) show the structure of the patients of both groups according to the assessment of the efficiency and effectiveness of the therapy. Although no statistically significant difference was found between the groups (ESWT-alone vs. ESWT-plus: *p* = 0.6362; *p* = 0.3049, respectively), the vast majority of patients assessed the therapy as very effective.

## 4. Discussion

Based on the results of our research, it can be seen that ESWT works better in conjunction with other treatment modalities. Although there were no significant differences between ESWT-alone and ESWT-plus therapy, ESWT-plus therapy resulted in clinically significant results as reported by 100% recovery in terms of pain and limitation to physical activity compared to 91% of ESWT-alone.

Extracorporeal shock wave therapy (ESWT) is increasingly used in orthopedics and sports medicine in the treatment of the lesions located within the musculoskeletal apparatus. Although the mechanism of action is not fully known, its positive effects in conditions resulting from overload are probably related to microdestruction [3,12,17]. Low level shock waves are known to cause various tissue responses and metabolic effects. It is presumed that the application of focused strokes causes microcracking of avascular tissues and of tissues poor in blood vessels, thus stimulating the revascularization process by releasing local growth factors and recruiting appropriate stem cells. The resulting changes increase joint mobility, prolong pain relief, and restore normal muscle tone [1,4,23,24].

In our study, after therapy, a significant reduction was noted in the intensity of pain experienced by patients during physical activity, assessed using the VAS scale and the Laitinen scale.

Krishnan et al. [25] showed positive reports that substantiate the effectiveness of ESWT on the treatment of plantar fasciitis by reporting the mean VAS scores to be decreased from an average of 9.2 to 3.4, at four weeks after treatment. In a study conducted on 60 patients, aged 45–68 years (mean age 55.6 years), Cosentino et al. [26] observed a reduction in pain intensity when awake, at rest, while walking, and during daily activities, measured using VAS, one month and three months after shock wave application. In addition, they also noted a reduction in the largest diameter of calcification in X-ray (> 1 mm). Similar results were not obtained in their control group. Similar results were obtained in Metzner’s study [27], which used ESWT on 63 patients with plantar aponeurosis inflammation. Each patient got 1000 impulses of ESWT; the stream density of the emitted energy was 0.35 mJ/mm^2^. The pain on VAS was examined 6 weeks, 18 months, and 72 months after the end of ESWT. The level of pain decreased, and an initial 30% of patients without pain increased to 81% of the patients after 6 weeks, 88% of the patients after 18 months, and 96% of the patients in the last examination 72 months after the end of ESWT. On the basis of the results, the authors concluded that the used ESWT doses successfully decreased the pain, and the treatment effects gave satisfying long-term results. In the study by Koch et al. [28], after the completion of therapy, a significant reduction in pain was also achieved, with the same satisfactory results (VAS and Laitinen scale) in the morning, during the day, and in the evening. The effects were achieved after the 5th ESWT treatment and remained one week after the end of treatment. Similar results were obtained in a study of the effectiveness of shock wave treatments in 22 patients with heel spur [29]. The patients underwent five treatment sessions, with four to six day breaks between sessions. The procedure was performed on the three most painful locations, which were detected manually. Significant reductions in pain were noted after therapy in the morning and at night, and the symptoms were significantly reduced after therapy, during the standing test, and during manual examination of the pain points in the foot. Moretti et al. [30] evaluated the analgesic efficacy of low doses of ESWT for foot plantar fasciitis in 54 runners-athletes. The subjects received a weekly shockwave of 1000 impulses with 0.06 mJ/mm^2^ energy density. The pain was assessed on VAS. ESWT treatment continued for four weeks, then the patients were examined after 45 days, and 6 and 24 months after the last session. The clinical results were excellent in 59% of cases, good in 12% of cases, satisfactory in 21%, and clearly unsatisfactory in 8%. The low-energy ESWT seems to be a good means to treat inflammation of foot plantar fascia in runners, because the resulting improvement persisted for 24 months from the end of ESWT. Additionally, Hammer et al. [31] assessed the analgesic efficacy of ESWT in 57 patients with painful chronic inflammation of the plantar fascia. Patients treated with ESWT were given 3000 impulses of shocks with energy density of 0.2 mJ/mm^2^ at weekly intervals. Two years after the end of treatment, the level of pain on a VAS scale in patients treated with ESWT decreased 94%.

In addition, in our study, therapy was found to have a positive effect on the symptoms experienced during walking and recreational activity, as indicated by the AOFAS score.

A previous study on ESWT on the level of pain and reduction of functional disorders in patients with plantar fasciitis and the accompanying heel spur yielded similar results [31]. The study compared the effects of ESWT alone with those of ESWT preceded by non-steroidal anti-inflammatory drugs and diclofenac iontophoresis. Pain complaints experienced by patients during rest, daily activities, and standing on one leg were assessed. The results indicate that the pain was significantly reduced after 12 weeks of ESWT; however, no significant differences were found between the two groups. Additionally, Samar G Soliman [32] assessed the efficacy of extracorporeal shock-wave therapy compared with local platelet-rich plasma injection for treatment of plantar fasciitis. This study included 60 patients, comprising 48 female and 12 male patients with plantar fasciitis diagnosed clinically and by ultrasound. Thirty patients received single local PRP injection and thirty patients received three sessions of ESWT weekly. VAS and AOFAS ankle-hind foot scale score in patients with calcaneal spur show more improvement in the ESWT group at 1 month after treatment (*p* = 0.019 and *p* = 0.009, respectively). Another study on patients undergoing ESWT also noted a significant reduction in pain at night, at rest, and under pressure, and an increase in the distance that the participants could walk without the need to rest [33]. Additionally, multiple meta-analyses of randomized controlled trial (RCTs) showed that ESWT decreases pain and improves function [21,31,34,35,36,37]. ESWT was found to yield significant improvements in all 20 tested patients suffering from pain in the joints of the feet in one study [4]. In addition, another study found ESWT to yield improvements in heel spurs in about 90% of cases: the patients experienced a great deal of relief when starting to walk, and then less problems when putting weight on the foot. Treatment also resulted in a strong analgesic effect immediately after the first application; however, a transient crisis may occur after the second or third treatment [5].

Chronic pain is a common problem in primary care that not only limits functions, but also adversely affects the quality of life of patients [38]. Therefore, the reduction of pain is a very important therapeutic target in the treatment of many diseases, including plantar fasciitis. In our study, the effect of extracorporeal shock wave therapy was confirmed as an effective and safe method of treating pain associated with plantar fasciitis.

Additionally, in the present study, the subjects from the ESWT-alone group demonstrated earlier effects than those who had received other procedures before therapy. It is possible that this difference may derive from assigning even a minimal improvement in the perception of pain to shock wave therapy, suppressing the effects of therapy by previously performed treatments. It may also be influenced by the habits of patients and their resulting higher expectations. This is our assumption, based on the available literature on the effectiveness of ultrasound and laser therapy. According to the literature [18], both ultrasound and laser therapy have an analgesic effect [39,40], so perhaps it was more difficult for these patients to isolate the analgesic effect of the shock wave. In addition, the study is based on the subjective feelings of patients, so we suspect that a possible reason for the later observation of improvement was the greater expectations of patients. Nevertheless, the effects were observed after each treatment.

Further clinical evidence confirming the positive effects of therapy in the treatment of orthopedic diseases continues to accumulate. In addition, the procedure is non-invasive and significantly reduces the need for orthopedic surgeries. In the literature, ESWT is also comparable to surgical plantar fasciotomy without surgical risk and gives good long-term results [41].

Strengths and limitations of the study. A key strength of our study is its relatively long five-year observation period which confirms that improvement lasted more than a few months. Moreover, all patients received the same type and dose of shock wave, and the power, frequency, and time between treatments did not differ from trial to trial, allowing us to assess the specific type of shockwave and the effect of a specific dose. Finally, the lack of local anesthesia during ESWT therapy increased the homogeneity between the compared trials. However, the presented study had some limitations. Firstly, the subgroups were quite small. The sample size should have been calculated, but this was not done, due to the small number of subjects who met all the eligibility criteria for the study. We selected 39 subjects; this was a necessary selection, as only these patients met the criterion “that patients did not receive any physical therapy prior to inclusion.” Secondly, although the patients were randomly assigned to the groups by a person from the research team who had no previous contact with the eligible patients, there is always a risk of bias, because this person had access to the data of all patients. In addition, the study did not analyze the potential mechanisms of the observed improvement, for example, by assessing blood supply improvement or structural changes using Doppler ultrasound. Ultrasound imaging was performed in the study to confirm the presence of plantar fasciitis, but the findings are based on the subjective assessment of patients and not objective tests, such as ultrasonography or blood flow. There were only two time points in the study at which patient information was collected: the first was before the extracorporeal shock wave therapy was performed (December 2015 to March 2016), and the second was five years after the procedure to check whether the pain had reappeared (December 2020 to March 2021). Therefore, important methodological limitation is the lack of other time points and diagnostic tests, within that 5-year follow-up period. Patient responses are mainly based on delayed recall, therefore the data obtained is subjective and may be inaccurate. As such, care should be taken when interpreting our findings.

## 5. Conclusions

Extracorporeal shock wave therapy is an effective form of therapy for plantar fasciitis experienced by amateur runners. Our observations also show that ESWT works better when combined with other treatments. These observations may be the starting point for further research on the effectiveness of the shock wave in combination with other treatments.

## Figures and Tables

**Table 1:** The structure of the treatment groups according to sex.

Group	N (%)	Sex		*p*-Level
		Male	Female	
ESWT-alone	N	8	15	0.1831
	%	34.78	65.22	
ESWT-plus	N	9	7	
	%	56.25	43.75	

No statistically significant difference was found between the groups in terms of sex (*p* = 0.1831).

**Table 2:** Characteristics of patients from the two treatment groups by age and BMI.

Variable	Measure	ESWT-Alone	ESWT-Plus	*p*-Level
Age	Mean ± SD	52.17 ± 9.49	56.75 ± 4.84	0.2628
	Me (IQR)	56 (48–60)	55.5 (52.5–61.5)	
	Min–Max	34–64	51–64	
BMI	Mean ± SD	28.51 ± 3.61	28.40 ± 4.46	0.6414
	Me (IQR)	28.41 (25.86–31.14)	28.06 (24.46–30.80)	
	Min–Max	20.05–36.26	23.44–37.13	

There were no statistically significant differences between the groups in terms of age and body mass index (respectively: *p* = 0.2628 and *p* = 0.6414).

**Table 3:** Structure of patients by BMI.

Group	N (%)	BMI				*p*-Level
		Normal	Overweight	Obesity I	Obesity II	
ESWT-Alone	N	3	14	4	2	0.2925
	%	13.04	60.87	17.39	8.70	
ESWT-plus	N	6	6	2	2	
	%	37.50	37.50	12.50	12.50	

**Table 4:** Assessment of pain intensity according to the VAS scale before and after therapy in the compared groups.

Group	Measure	Before Therapy	After Therapy	Effect Size	*p*-Level (before vs. after)
ESWT-alone	Me (IQR)	8 (7–10)	2 (1–3)	0.8384	<0.0001
	Min–Max	6–10	0–6		
ESWT-plus	Me (IQR)	8 (7.5–9.5)	1.5 (1–2)	0.8797	<0.0001
	Min–Max	5–10	1–4		

*p*-level (group comparison): before therapy: 0.9809; after therapy: 0.9200.

**Table 5:** Assessment of pain intensity according to the Laitinen scale before and after therapy in the compared groups.

Group	Measure	Before Therapy	After Therapy	Effect Size	*p*-Level (before vs. after)
ESWT-alone	Me (IQR)	10 (7–12)	2 (0–3)	0.8402	<0.0001
	Min–Max	6–13	0–6		
ESWT-plus	Me (IQR)	9 (7.5–10)	1 (0–2)	0.8860	<0.0001
	Min–Max	7–11	0–3		

*p*-level (group comparison): before therapy: 0.8840; after therapy: 0.9687.

**Table 6:** Assessment of the AOFAS index (total score) before and after therapy in the compared groups.

Group	Measure	Before Therapy	After Therapy	Effect Size	*p*-Level (before vs. after)
ESWT-alone	Me (IQR)	63 (48–80)	90 (83–100)	0.8351	<0.0001
	Min–Max	22–90	61–100		
ESWT-plus	Me (IQR)	66 (44.5–90)	90 (86.5–100)	0.8484	<0.0001
	Min–Max	30–90	76–100		

*p*-level (group comparison): before therapy: 0.9645; after therapy: 0.8380.

**Table 7:** Assessment of the AOFAS index (pain points) before and after therapy in the compared groups.

Group	Measure	Before Therapy	After Therapy	Effect Size	*p*-Level (before vs. after)
ESWT-alone	Me (IQR)	20 (20–20)	40 (30–40)	0.8473	<0.0001
	Min–Max	0–30	20–40		
ESWT-plus	Me (IQR)	20 (10–30)	40 (40–40)	0.8551	0.0001
	Min–Max	0–30	30–40		

*p*-level (group comparison): before therapy: 0.9535; after therapy: 0.7676.

**Table 8:** Assessment of the AOFAS index (function points) before and after therapy in the compared groups.

Group	Measure	Before Therapy	After Therapy	Effect Size	*p*-Level (before vs. after)
ESWT-alone	Me (IQR)	38 (33–50)	50 (41–50)	0.7054	0.0001
	Min–Max	19–50	35–50		
ESWT-plus	Me (IQR)	41 (31.5–50)	50 (42.5–50)	0.6903	0.0013
	Min–Max	23–50	39–50		

*p*-level (group comparison): before therapy: 0.9396; after therapy: 0.9574.

## Data Availability

The data underlying this article cannot be shared publicly for the privacy of individuals that participated in the study.

## Supplementary Materials

The following supporting information can be downloaded at: https://www.mdpi.com/article/10.3390/jcm11236926/s1, Table S1. The time that the first treatment effects were noted according to treatment group; Table S2. Patient fitness assessment according to treatment group; Table S3. Patient assessment of the effectiveness of therapy according to treatment group.
