# Supplementary material for: Long Term Effectiveness of ESWT in Plantar Fasciitis in Amateur Runners
Source: J Clin Med. 2022 Nov 24;11(23):6926. doi: 10.3390/jcm11236926 (PMC9737564; doi:10.3390/jcm11236926)
Supplement: Supplementary file 1 [file jcm-11-06926-s001.zip › jcm-2061225-supplementary.pdf]

Supplementary Materials

Table S1. The time that the first treatment effects were noted according to treatment group.

Group	N (%)	First effect					*p*-Level
		No Effect	After the First	After the Second	After the Third	After the Fourth	
ESWT-alone	N	2	16	4	0	1	0.0190
	%	8.70	69.57	17.39	0.00	4.35	
ESWT-plus	N	0	5	6	2	3	
	%	0.00	31.25	37.50	12.50	18.75	

Table S2. Patient fitness assessment according to treatment group.

Group	N (%)	Do You Feel More Physically Fit?		*p*-Level
		Yes	I Don't Know	
ESWT-alone	N	21	2	0.6362
	%	91.30	8.70	
ESWT-plus	N	16	0	
	%	100.00	0.00	

Table S3. Patient assessment of the effectiveness of therapy according to treatment group.

Group	N (%)	Evaluation of the Effectiveness of Therapy			*p*-Level
		Very Effective	Effective	Hard to Say	
ESWT-alone	N	17	4	2	0.3049
	%	73.91%	17.39%	8.70%	
ESWT-plus	N	12	4	0	
	%	75.00%	25.00%	0.00%	
